# Peripheral hearing sensitivity is similar between the sexes in a benthic turtle species despite the larger body size of males

**DOI:** 10.1002/ece3.70130

**Published:** 2024-08-08

**Authors:** Tongliang Wang, Jinxia Yang, Jinhong Lei, Jingdeng Huang, Haitao Shi, Jichao Wang

**Affiliations:** ^1^ Ministry of Education Key Laboratory for Ecology of Tropical Islands, Key Laboratory of Tropical Animal and Plant Ecology of Hainan Province, College of Life Sciences Hainan Normal University Haikou China

**Keywords:** adaptation, habitat, hearing sensitivity, *Pelodiscus sinensis*, sexual dimorphism

## Abstract

Sexually dimorphic hearing sensitivity has evolved in many vertebrate species, and the sex with a larger body size typically shows more sensitive hearing. However, generalizing this association is controversial. Research on sexually dimorphic hearing sensitivity contributes to an understanding of auditory sense functions, adaptations, and evolution among species. Therefore, the hypothesized association between body size and hearing needs further validation, especially in specific animal groups. In this study, we assessed hearing sensitivity by measuring auditory brainstem responses (ABRs) in both sexes of 3‐year‐old Chinese softshell turtles (*Pelodiscus sinensis*). In this species, male bodies are larger than those of female, and individuals spend most of their lives in the mud at the bottom of freshwater habitats. We found that for both sexes, the hearing sensitivity bandwidth was 0.2–0.9 kHz. Although males were significantly larger than females, no significant differences in ABR thresholds or latencies were found between males and females at the same stimulus frequency. These results indicate that *P. sinensis* hearing is only sensitive to low‐frequency (typically <0.9 kHz) sound signals and that sexually dimorphic hearing sensitivity is not a trait that has evolved in *P. sinensis*. Physiological and environmental reasons may account for *P. sinensis* acoustic communication via low‐frequency sound signals and the lack of sexually dimorphic hearing sensitivity in these benthic turtles. The results of this study refine our understanding of the adaptation and evolution of the vertebrate auditory system.

## Graphical abstract

In this study, we assessed sex‐based differences in body size and hearing sensitivity in Chinese softshell turtles (*Pelodiscus sinensis*). Studies on chelonian hearing are limited. We believe that our study makes a significant contribution to the literature because, although an association because body size and sexually dimorphic differences in auditory sensitivity of vertebrates has been reported, we found no such association in this study. Our results provide additional data to better understand the evolution and adaptation of vertebrate auditory systems.

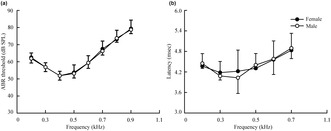

## 1 INTRODUCTION

Sexual dimorphism refers to differences such as size, shape, and color between females and males of the same species. These differences have evolved in almost all animals and represent an important area for research on phenotypic evolution (Cueva del Castillo et al., 2024; dos Santos et al., 2022; Juarez et al., 2023; Manoli et al., 2013; Pintore et al., 2023). Studies on sexual dimorphism are important for understanding the function, adaptive significance, and evolution of differences in the morphology, physiology, and behavior of organisms (Beltrán et al., 2022; Gayford, 2023; Ludwig et al., 2023; Shen et al., 2011; Shogren et al., 2022). However, research on sexual dimorphism has focused predominantly on body size or shape, while sexually dimorphic behaviors such as acoustic communication have been largely neglected.

Acoustic communication plays a role in almost all animal life histories, and hearing sensitivity can directly affect the efficiency of acoustic signal transmission (Aleksandrov & Dmitrieva, 1992; Chen & Wiens, 2020; Gerhardt & Huber, 2002; Köppl et al., 2014). Sexually dimorphic hearing sensitivity has evolved in vertebrates, including amphibians (Cobo‐Cuan et al., 2023; Schrode et al., 2014; Wang, Jia, et al., 2019; Zhu et al., 2022), reptiles (Wang, Li, et al., 2019), birds (Gall & Lucas, 2010), and rodents (Lin et al., 2022). In these species, females are usually larger than males; thus, sex‐related differences in hearing may be a consequence of differences in body size. Specifically, the sex with the larger body size is typically associated with more sensitive hearing. However, some studies have drawn contradictory conclusions (Hetherington, 1994; Shen et al., 2011). Based on these discrepancies, additional research on the sexual dimorphism of hearing sensitivity in species with males exhibiting larger body sizes than females is needed to understand auditory sense function, adaptation, and evolution in vertebrates.

As an ancient living reptile group, chelonians have long been considered to be non‐vocal, relatively few studies have addressed acoustic communication in this group. Recent studies have shown that turtles can produce low‐frequency sounds during hatching (de Melo et al., 2023; Monteiro et al., 2019), nesting (Ferrara et al., 2014), and mate choice or sexual selection (Galeotti et al., 2005; Pellitteri‐Rosa et al., 2011). Remarkably, some species of turtle have a specific auditory sense (usually below 1.0 kHz) that allows for the perception of acoustic signals in the air or underwater (Christensen‐Dalsgaard et al., 2012; Wang et al., 2021). Our previous research showed that turtle vocalizations differ by sex (Zhou et al., 2022; Zhou, Lei, Zhai, Shi, & Wang, 2023) and that female *Trachemys scripta elegans*, which have a larger body size than males, have more sensitive hearing than males (Wang, Li, et al., 2019). Furthermore, although the patterns differ, almost all turtles display sexual size dimorphism; for example, females are larger than males in some species but smaller in others (Berry & Shine, 1980; Gibbons & Lovich, 1990; Lichtig & Lucas, 2023; Moskovits, 1988). Therefore, turtles are an ideal animal group for studying the relationship between hearing sensitivity and body size sexual dimorphism, and studies on this group will provide further insights into the adaptation and evolution of acoustic communication in vertebrates.

The Chinese softshell turtle (*Pelodiscus sinensis*) is a freshwater turtle. Compared with many other turtle species, *P. sinensis* shows a sex‐dependent dimorphic growth pattern, with males exhibiting more rapid growth and larger body sizes (Zhou & Zhu, 2011). These turtles spend most of their lives in the mud at the bottom of the water and prefer to feed in the dark (Li et al., 2020; Zhou et al., 1998). As an ideal model of turtles, many studies have been conducted on the ecology (Zhu et al., 2021), physiology (Li et al., 2023; Zhong et al., 2020), and molecular biology (Lei et al., 2022; Wang et al., 2023) of this species, and research on its vocal behaviors has demonstrated highly diverse underwater vocalizations (i.e., 10 caLL types with low frequencies under 750 Hz and high frequencies of approximately 15,000 Hz) and a tendency for vocalization to become more diverse with age (Zhou, Lei, Zhai, Lu, et al., 2023). However, its auditory characteristics have not been studied. This gap in auditory research on *P. sinensis* has limited our understanding of the functional differences in hearing between male and female turtles with an unusual sexual size dimorphic pattern and unique habitat.

In this study, the hearing sensitivity bandwidth, auditory brainstem response (ABR) threshold, and latency were measured in both sexes of *P. sinensis* to study their hearing responses and determine whether males with larger body sizes display more sensitive hearing than females. Given that males have a larger body size than females, we predicted that hearing in males would be more sensitive than that in females.

## 2 MATERIALS AND METHODS

### 2.1 Experimental animals

All *P. sinensis* specimens were purchased from farms in Hainan Province and maintained in aquaria (length × width × height: 2.0 × 1.0 × 0.25 m; water depth: 0.15 m) under natural conditions for approximately 2 weeks, with four individuals in each tank. In addition, a round pelleted artificial diet with a diameter of approximately 6.0 mm (Dolphin Aquarium Co., Ltd., Guangdong, China) was provided ad libitum. Before electrode placement, each individual was deeply anesthetized using a solution of 5% chloral hydrate (CAS No. 302‐17‐0; Tianjin Damao Chemical Reagent Factory, Tianjin, China) dissolved in distilled water. The anesthetic was administered via intraperitoneal injection at an initial dose of 0.006 mL/g. Additional doses (20% of the initial dose) were administered when an individual was not sufficiently anesthetized. Electrophysiological experiments commenced when the subject showed no reflexive response to stimulation of the hind leg muscles and eyes with a pair of forceps (Wang et al., 2022). The animals remained relatively motionless for more than 150 min. The animal treatment procedures were approved by the Animal Research Ethics Committee of the Hainan Provincial Education Centre for Ecology and Environment, Hainan Normal University (HNECEE‐2018‐001), and carried out in strict compliance with the guidelines of the institution.

### 2.2 Measurement of morphological data

The body mass of *P. sinensis* was recorded using an electronic balance (0.1 g precision; SE‐3001FZH; Ohaus Instrument Co., Ltd., Shanghai, China). Carapace length was measured using a Mitutoyo 500–153 digital caliper (0.01 mm resolution; Mitutoyo Corp., Kawasaki‐shi, Japan).

### 2.3 Measurement of auditory brainstem responses

All recording ABRs were performed in a soundproof booth lined with echo‐attenuating acoustic foam. Prior to recording ABRs, ABR stimulus levels were calibrated (Wang et al., 2021). Stimulus presentation, ABR acquisition, equipment control, and data management were coordinated using a TDT RZ6 Multi‐I/O Processor (Tucker‐Davis Technologies, Alachua, FL, USA). Based on our previous studies (Wang et al., 2021; Wang, Li, et al., 2019), two types of signals were used as acoustic stimuli (tone bursts and broadband clicks) in this study (Brittan‐Powell et al., 2002; Wang et al., 2022). Tone burst (9 ms duration, 2 ms rise time, 2 ms fall time, and with a sample rate of 24,414 Hz and alternating polarity) was synthesized digitally from 0.2 to 1.5 kHz in 100 Hz increments, attenuated in 5 dB steps from 85 to 35 dB sound pressure level (dB SPL re 20 μPa), and presented at a rate of 4/s. Clicks were 0.1 ms in duration with a 249 ms interstimulus interval, attenuated in 5‐dB steps from 90 to 35 dB SPL, and presented at a rate of 4/s (Wang, Li, et al., 2019). Each ABR wave represented the average response to 200 stimuli. The signals from the electrodes were amplified (20×) and filtered (high pass: 30 Hz; low pass: 3 kHz; notch filtered: 50 Hz). The sound stimuli were played once from low to high frequency (Wang, Li, et al., 2019). ABRs were recorded for approximately 100 min, during which time the animal remained anesthetized.

Threshold measurements were defined as the lowest stimulus level, below which no repeatable responses could be recognized by visual inspection. To reduce interrater variability, all individual ABR thresholds were confirmed by two trained observers (Wang et al., 2022). ABR latencies were recorded as the time from stimulus onset to the first negative waveform valley.

### 2.4 Data analysis and statistics

The ABR thresholds and latencies obtained from female and male *P. sinensis* in response to tone stimuli were sorted and analyzed using SPSS software (version 22.0; IBM Corp., Chicago, IL, USA). Prior to statistical analysis, all data were examined for assumptions of normality and homogeneity of variance using the Shapiro–Wilk and Levene tests, respectively. Data on ABR thresholds and latencies in both sexes were analyzed using repeated‐measures factorial ANOVA and one‐way ANCOVA. The body mass and carapace length both sexes were analyzed using one‐way ANCOVA. Results are presented as means ± standard deviations, and differences with *p*‐values < .05 were considered statistically significant.

## 3 RESULTS

### 3.1 Morphological data

Data related to body mass (males = 1103.1 ± 64.6 g, females = 862.8 ± 30.0 g) and carapace length (males = 199.3 ± 4.9 mm, females = 179.2 ± 5.7 mm) are presented in Table 1. Both body mass and carapace length were significantly larger in males than in females (*p* < .001).

**TABLE 1:** Differences in the morphological traits of *Pelodiscus sinensis* between the sexes.

Parameters	Sex		Statistical summary	
	Female (*n* = 8)	Male (*n* = 8)	*F*	*p*
Body mass (g)	862.8 ± 30.0	1103.1 ± 64.6	91.17	<.001
Carapace length (mm)	179.2 ± 5.7	199.3 ± 4.9	57.18	<.001

### 3.2 ABR wave morphology

**FIGURE 1:**
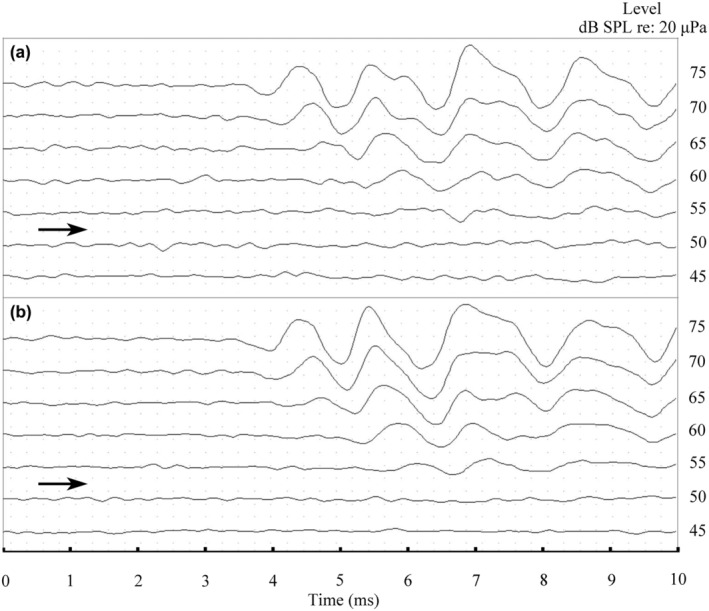
Auditory brainstem responses (ABRs) as a function of stimulus intensity were evoked by a tone burst of 0.3 kHz in a female (a) and a male (b) *Pelodiscus sinensis*. The right pointing arrowheads indicate the visually detected thresholds for stimulus intensity.

In both females (Figure 1a) and males (Figure 1b), the ABRs to tone‐burst stimuli were characterized by valley–peak waveforms. However, the waveforms in both sexes were not obvious below 55 dB with a tone stimulus of 0.3 kHz.

### 3.3 Hearing sensitivity bandwidth and ABR threshold

**FIGURE 2:**
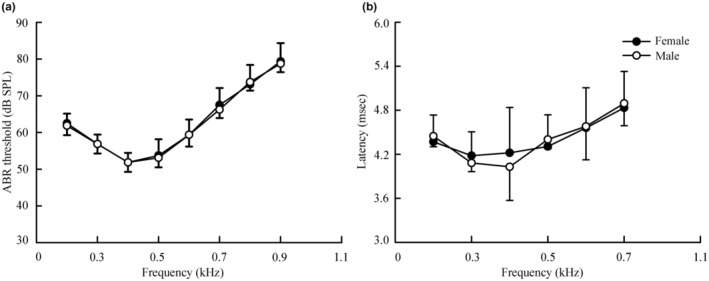
Auditory brainstem response (ABR) threshold (a) and latency (b) in female and male *Pelodiscus sinensis*.

Hearing sensitivity bandwidth refers to the frequency range of sound signals received by animals. The hearing sensitivity bandwidth was 0.2–0.9 kHz in both female and male *P. sinensis* (Figure 2a).

ABR thresholds were measured in individual turtles at predetermined stimulus frequencies. Significant differences were observed in the ABR thresholds and frequencies (*F* = 284.70, *df* = 2.54, *p* < .01), whereas no significant interaction effects were not observed between sex and frequency (*F* = 0.26, *df* = 2.54, *p* = .82). There were no significant differences in ABR thresholds by sex (*F* = 0.06, *df* = 1, *p* = .82). The ABR thresholds for each stimulus frequency were compared between female and male *P. sinensis* and they did not show significant differences at the same stimulus frequency (*p* > .05) (Table 2).

**TABLE 2:** Differences in the auditory brainstem response (ABR) threshold for tone stimulus at each stimulus frequency of *Pelodiscus sinensis* between the sexes.

Frequency (kHz)	Sex		Statistical summary	
	Female	Male	*F*	*p*
0.2	62.5 ± 2.7	61.9 ± 2.6	0.23	.64
0.3	56.9 ± 2.6	56.9 ± 2.6	0.00	1.00
0.4	51.9 ± 2.6	51.9 ± 2.6	0.00	1.00
0.5	53.8 ± 4.4	53.1 ± 2.6	0.12	.74
0.6	59.4 ± 4.2	59.4 ± 3.2	0.00	1.00
0.7	67.5 ± 4.6	66.3 ± 2.3	0.47	.51
0.8	73.1 ± 5.3	73.8 ± 2.3	0.09	.76
0.9	79.4 ± 5.0	78.8 ± 2.3	0.10	.75

### 3.4 Latency

Latencies were measured between stimulus onset and waveform valley. To determine the latency of all frequencies of the hearing sensitivity bandwidth (from 0.2 to 0.9 kHz), an ABR waveform of 75 dB was chosen for each stimulus frequency (Figure 2b). There were significant differences in latency and frequency (*F* = 22.48, *df* = 2.28, *p* < .01), whereas there was no significant interaction between sex and frequency (*F* = 0.94, *df* = 2.28, *p* = .41). There were also no significant differences in latency by sex (*F* = 0.01, *df* = 1, *p* = .97). As the stimulus frequency (0.2–0.7 kHz) increased, the ABR latencies lengthened, but no significant difference in ABR latency at the same stimulus frequency was found between female and male *P. sinensis* (*p* > .05) (Table 3).

**TABLE 3:** Differences in the latency for tone stimulus at each stimulus frequency of *Pelodiscus sinensis* between the sexes.

Frequency (kHz)	Sex		Statistical summary	
	Female	Male	*F*	*p*
0.2	4.37 ± 0.36	4.45 ± 0.15	0.31	.59
0.3	4.18 ± 0.32	4.08 ± 0.12	0.67	.43
0.4	4.22 ± 0.62	4.03 ± 0.46	0.50	.49
0.5	4.31 ± 0.43	4.40 ± 0.13	0.37	.55
0.6	4.56 ± 0.54	4.58 ± 0.46	0.01	.94
0.7	4.83 ± 0.49	4.90 ± 0.30	0.09	.77

## 4 DISCUSSION

Chelonians occupy a wide range of ecological niches from deserts to oceans (Berry & Shine, 1980; Germano, 2014; Lutz et al., 2002). Studies on hearing sensitivity and sexual dimorphism in *P. sinensis*, which prefers living in mud at the bottom of a water body, have become increasingly important because they indicate the adaptation of turtle auditory systems to special habitats and the evolution of vertebrate auditory systems. Contrary to our predictions, although the body size of male *P. sinensis* was significantly larger than that of female *P. sinensis*, our results did not reveal sexually dimorphic hearing sensitivity in this species.

The study of acoustic communication in turtles is limited compared to that in other reptile species. Studies have shown that tokay geckos (*Gekko gecko*) and green anoles (*Anolis carolinensis*) are sensitive to frequencies of approximately 5 kHz (Brittan‐Powell et al., 2010), whereas sea snakes (*Hydrophis stokesii*) are sensitive to low‐frequency sounds (40 Hz up to 600 Hz) (Chapuis et al., 2019). In the present study, we found that *P. sinensis* is sensitive to sounds below 0.9 kHz. This was similar to findings for the green sea turtles *Chelonia mydas* (0.05–0.8 kHz) (Piniak et al., 2016), loggerhead sea turtle *Caretta caretta* (0.25–0.75 kHz) (Bartol et al., 1999), and red‐eared slider *T. scripta elegans* (below 1.4 kHz) (Wang, Li, et al., 2019). Our results provide further confirmation that the auditory systems of chelonians are sensitive only to low‐frequency sound signals (mostly below 1 kHz). These results suggest that reptiles may have evolved diverse hearing characteristics as an adaptation to different acoustic environments.

Anatomical evidence suggests that there are at least two distinct areas in lizard auditory papillae that respond best to frequencies below 1 kHz and higher (Manley, 2000). The auditory systems in gekkonids are sensitive to high frequencies because the basilar papillae are well‐developed (Manley, 2002; Manley et al., 1999). This could explain why the *G. gecko* auditory system responds to high‐frequency sound signals. Anatomical studies have verified that the auditory papillae of testudinates are small and simple, present only one kind of sensory cell, and only respond to low sound frequencies (<1 kHz) (Crawford & Fettiplace, 1980). Therefore, the sensitivity of *P. sinensis* to low‐frequency sound signals is associated with the small auditory papillae in its auditory system.

Sexually dimorphic hearing sensitivity has evolved in many vertebrate species. Numerous studies have shown that female frogs and toads have more sensitive auditory systems than males (Schrode et al., 2014; Wang et al., 2016; Wang, Jia, et al., 2019; Yang et al., 2019; Zhu et al., 2022). This conclusion is similar to that of our previous study on other turtle species (Wang, Li, et al., 2019) and may be related to the larger bodies of females. However, studies have also revealed that although male frogs have a smaller body size, their hearing is more sensitive than that of females (Liu et al., 2014; Shen et al., 2011). Moreover, despite the smaller sample size, female Steller's sea lions (*Eumetopias jubatus*) have a smaller body size and greater hearing sensitivity (Kastelein et al., 2005). In this study, we found that although the body size of male *P. sinensis* was significantly larger than that of female *P. sinensis*, sexually dimorphic hearing sensitivity did not occur in this species. To our knowledge, this is the first study to verify the absence of dimorphic sexual hearing sensitivity in a turtle species.

Sex differences in hearing responses are largely associated with sexually dimorphic auditory organs, such as middle ear ossicles (Feng et al., 2006; Shen et al., 2011), tympanic membranes (Feng et al., 2006; Mason et al., 2003; Shen et al., 2011), or auditory papillae (Wilczynski, 1986). In the present study, although the shape of auditory organs was not compared between the sexes of *P. sinensis*, the conclusions from the above studies suggest that the size or mass of these organs was not significantly different between the male and female of *P. sinensis*. Moreover, although hearing sensitivity based on sex differences may be associated with differences in body size, females in many species are larger than males (Bass et al., 2016). Sexually dimorphic hearing sensitivity in *T. scripta elegans*, for example, does not appear to be related to differences in the size of tympanic membranes (Wang, Li, et al., 2019). Furthermore, no significant differences were detected in the scaling of bony middle ear cavity volumes and head size in chelonian families (Willis et al., 2013). Our results verified that sexually dimorphic body size was not directly related to hearing sensitivity in *P. sinensis*.

Previous studies found that frogs have evolved diverse broadband signals and high‐frequency hearing sensitivity as an evolutionary adaptation to noisy environments (Feng et al., 2006; Liu et al., 2014; Shen et al., 2011; Zhao et al., 2017). Research on the ecology of *P. sinensis* has indicated that these turtles spend most of their lives in the mud at the bottom of freshwater bodies and prefer to feed in the dark (Li et al., 2020; Zhou et al., 1998). High habitat consistency between female and male *P. sinensis* may explain why sexually dimorphic hearing sensitivity did not evolve in these benthic turtles.

Overall, our results suggest that the *P. sinensis* auditory system is sensitive only to low‐frequency sound signals (typically below 0.9 kHz) and that sexually dimorphic hearing sensitivity has not evolved in this species. Small auditory papillae may limit *P. sinensis* acoustic communication to low frequencies. Remarkably, Zhou, Lei, Zhai, Lu, et al. (2023) reported that *P. sinensis* can emit high‐frequency vocalizations (>15,000 Hz) (Zhou, Lei, Zhai, Lu, et al., 2023), although the frequency of these signals was not in their range of hearing. According to the matched filter hypothesis, receiving systems are thought to have co‐evolved with acoustic communication signals (Gerhardt & Huber, 2002; Ryan & Wilczynski, 1988); however, additional behavioral or physiological research is required to answer the question of why the auditory system of *P. sinensis* does not respond to the high‐frequency sounds emitted by this species. Our study contributes to the body of research on hearing sensitivity in other groups of reptiles such as geckos, which show high‐frequency communication in terrestrial environments, and sea snakes, which show low‐frequency communication in oceanic environments, by defining the particularities of the auditory systems of a benthic fresh water turtle species and providing insights into the adaptation and evolution of vertebrate auditory systems.

## AUTHOR CONTRIBUTIONS

**Tongliang Wang:** Funding acquisition (equal); writing – original draft (equal); writing – review and editing (equal). **Jinxia Yang:** Data curation (equal). **Jinhong Lei:** Data curation (equal). **Jingdeng Huang:** Data curation (equal). **Haitao Shi:** Supervision (equal). **Jichao Wang:** Supervision (equal); writing – review and editing (equal).

## FUNDING INFORMATION

This work was supported by the Natural Science Foundation of Hainan Province under Grant number 320QN256; High‐level Talent Project of the Natural Science Foundation of Hainan Province under Grant number 322RC661; and Specific Research Fund of the Innovation Platform for Academicians of Hainan Province.

## CONFLICT OF INTEREST STATEMENT

The authors declare no competing interests.

## Supporting information


Data S1:

## Data Availability

The data are available in Data S1.
